# Noonan syndrome caused by RIT1 gene mutation: A case report and literature review

**DOI:** 10.3389/fped.2022.934808

**Published:** 2022-09-07

**Authors:** Ping Zha, Ying Kong, Lili Wang, Yujuan Wang, Qing Qing, Liying Dai

**Affiliations:** Department of Neonatology, Anhui Provincial Children's Hospital, Hefei, China

**Keywords:** Noonan syndrome, *RIT1*, variant locus, clinical phenotype, hypertrophic cardiomyopathy

## Abstract

**Objective:**

Noonan syndrome (NS), an autosomal dominant disease known as a RASopathy, is caused by germline mutations in mitogen-activated protein kinase pathway genes. A *RIT1* gene mutation has been found to cause NS. The present study summarizes *RIT1* gene mutation sites and associated clinical phenotypes.

**Methods:**

We retrospectively analyzed the clinical characteristics of a case of NS caused by *RIT1* mutation in our hospital, and searched the PubMed database, China National Knowledge Infrastructure (CNKI) database and Wanfang database with the keywords Noonan syndrome and *RIT1*. Studies published between May 1, 2014 and July 1, 2021 were retrieved. By reviewing the abstracts and full text of the studies, we screened NS cases associated with *RIT1* mutation in children 0–18 years of age. The clinical characteristics of these cases were summarized.

**Results:**

A total of 41 cases were analyzed, including 13 boys and 28 girls. There were 14 premature cases. The age at diagnosis was 4 days to 18 years, and 10 cases were diagnosed at 0–1 years of age. Common amino acid substitution positions included 57 (13/41), 95 (7/41), 82 (8/41), and 90 (4/41). A total of 63.63% cases had abnormal prenatal examination results, manifesting mainly as fetal neck edema, polyhydramnios and cardiac malformation. With respect to abnormal conditions after birth, 70–80% of patients had typical developmental malformations of the face, neck and thorax; 19/35 patients had abnormal lymphatic development; and a portion of patients had short stature and motor development disorders. A total of 87.80% (36/41) patients had cardiac dysplasia, among which hypertrophic cardiomyopathy (HCM) accounted for 58.53%. A total of 84.62% of patients carrying the p.A57G mutation had HCM, but no HCM was found in patients with the p.G95A mutation. A total of 34.15% of patients had pulmonary artery or pulmonary valve stenosis (PVS). In patients with the p.M90I mutation, 75% had PVS. Patients with concurrent HCM and PVS accounted for 19.51 and 48.78% of patients had supraventricular tachycardia.

**Conclusion:**

A *RIT1* gene mutation causing NS was associated with a high rate of abnormal prenatal examination findings. Most patients had typical NS craniofacial deformities, and some have short stature and motor development disorders. The cardiac deformity rate was high, and HCM was common. Some patients had supraventricular arrhythmias. Heart abnormalities showed high heterogeneity, given the various mutation loci.

## Introduction

Noonan syndrome (NS, OMIM 163950) is an autosomal dominant disorder that has an incidence rate of 1/1,000–1/2,500 (1), and is hereditary and sporadic (2). Patients have typical features of craniofacial deformities, postnatal growth retardation and congenital heart defects. A variety of gene mutations have been associated with this condition (3). *RIT1* gene mutation has been found to cause NS; however, the reported cases remain limited. In this study, we analyzed the clinical data of a child with *RIT1* gene mutation related NS who was admitted to our hospital, and reviewed the relevant studies in the literature. We summarized the clinical phenotypes and the variations in the heart abnormalities associated with various gene mutation loci. This study provided a clinical basis for the management of *RIT1* mutation related NS.

## Research methods

The clinical data of a case of NS caused by *RIT1* gene mutation were retrospectively analyzed, and related studies were reviewed.

### Case data

A 3-day-old boy was admitted to our hospital because of difficulty breathing and bruising for 3 days after birth. The patient was G_4_P_2_, his gestational age was 39^+3^ weeks, and his birth weight was 3,420 g. His APGAR score was normal. His mother had polyhydramnios during pregnancy. His parents and older brother are healthy. Admission signs included right upper eyelid ptosis, low-set ears, right pleural effusion, right cryptorchidism and left testicular hydrocele. Echocardiography after admission revealed an atrial septal defect (foramen secundum 0.36 cm) and pulmonary hypertension. The child was fed deep hydrolyzed milk powder, and was administered anti-infection, oxygen inhalation and other treatments after birth. The child was discharged after his dyspnea improved. After obtaining informed consent from the child's parents, we collected peripheral blood samples from the child and his parents and sent them to the Chigene (Beijing) Translational Medical Research Center Co. Ltd for gene sequencing. A point mutation (c.170C>G p.A57G) was identified in the *RIT1* gene. The child was diagnosed with NS. Because the mutation was not detected in the child's parents, and the parents had normal phenotypes, the mutation was determined to be a *de novo* heterozygous mutation, in line with the pathogenesis of autosomal dominant diseases. The risk of NS in the second child was low. The mutation is not listed in the Human Gene Mutation Database, Online Mendelian Inheritance in Man, Clinvar or several other common genetic variation databases (DYDF, dbSNP, 1000 Genomes Project and ExAC). The child developed dyspnea again at the age of 1.5 months. Ultrasound was performed again and showed bilateral pleural effusion, and echocardiography showed a ventricular septal hypertrophy 1.5 cm thick in the middle, which was accompanied by increased left ventricular outflow tract flow velocity. The child was admitted again and diagnosed with chylothorax. He subsequently underwent right thoracic drainage and was fed deep hydrolyzed milk powder until his symptoms improved. At a follow-up at the age of 6 months, right cryptorchidism was identified by ultrasound, and surgery for his undescended testicle was performed. During the operation, the right testis was found to be dysplastic, with a size of 0.6 × 0.6 cm, and was fixed in the scrotum by surgery. Echocardiography at the age of 7 months showed that the ventricular septal hypertrophy had disappeared, his pulmonary artery flow velocity increased, and the foramen ovale was patent. At examination at the age of 1.5 years, the child's height was 79 cm, and his weight was 9.5 kg. He had fine, curly hair, slightly delayed language development and normal motor function.

### Literature search and screening

We searched the PubMed database, CNKI database and Wanfang database for studies published from May 1, 2014 to July 1, 2021, with the key words Noonan syndrome and *RIT*1. By reviewing the abstract and full text, we screened studies reporting Noonan syndrome cases 0–18 years of age and related to *RIT1* gene mutations.

## Results

### General information

**Figure 1:**
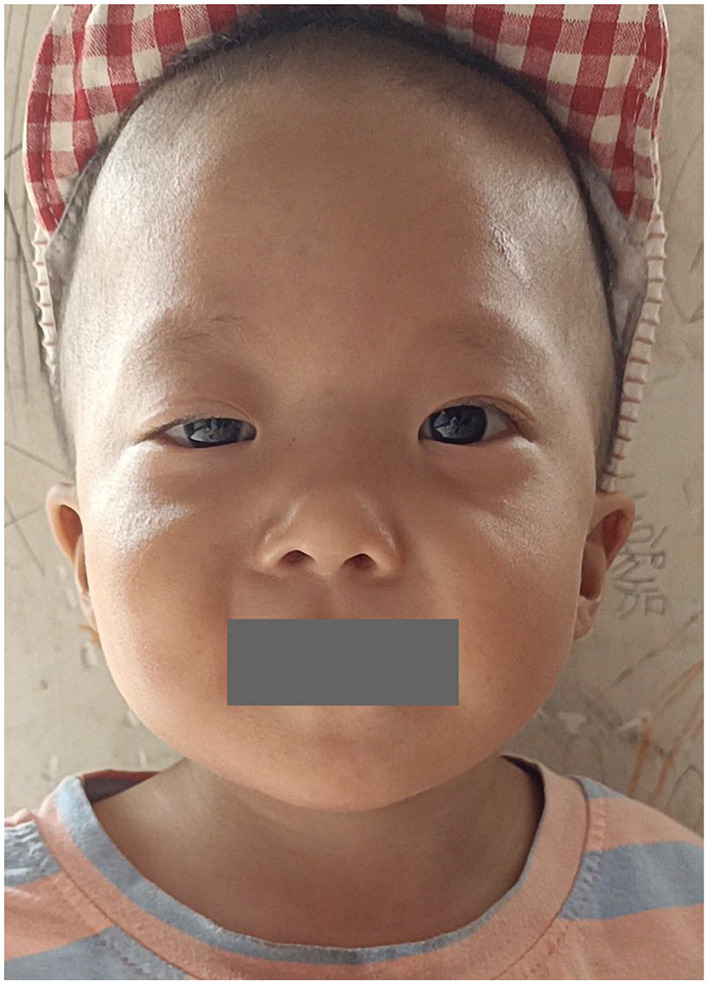
Photograph of the child with *RIT1* mutation related NS at 1.5 years of age. The facial characteristics include fine soft hair, low-set ears and right upper eyelid ptosis.

**Figure 2:**
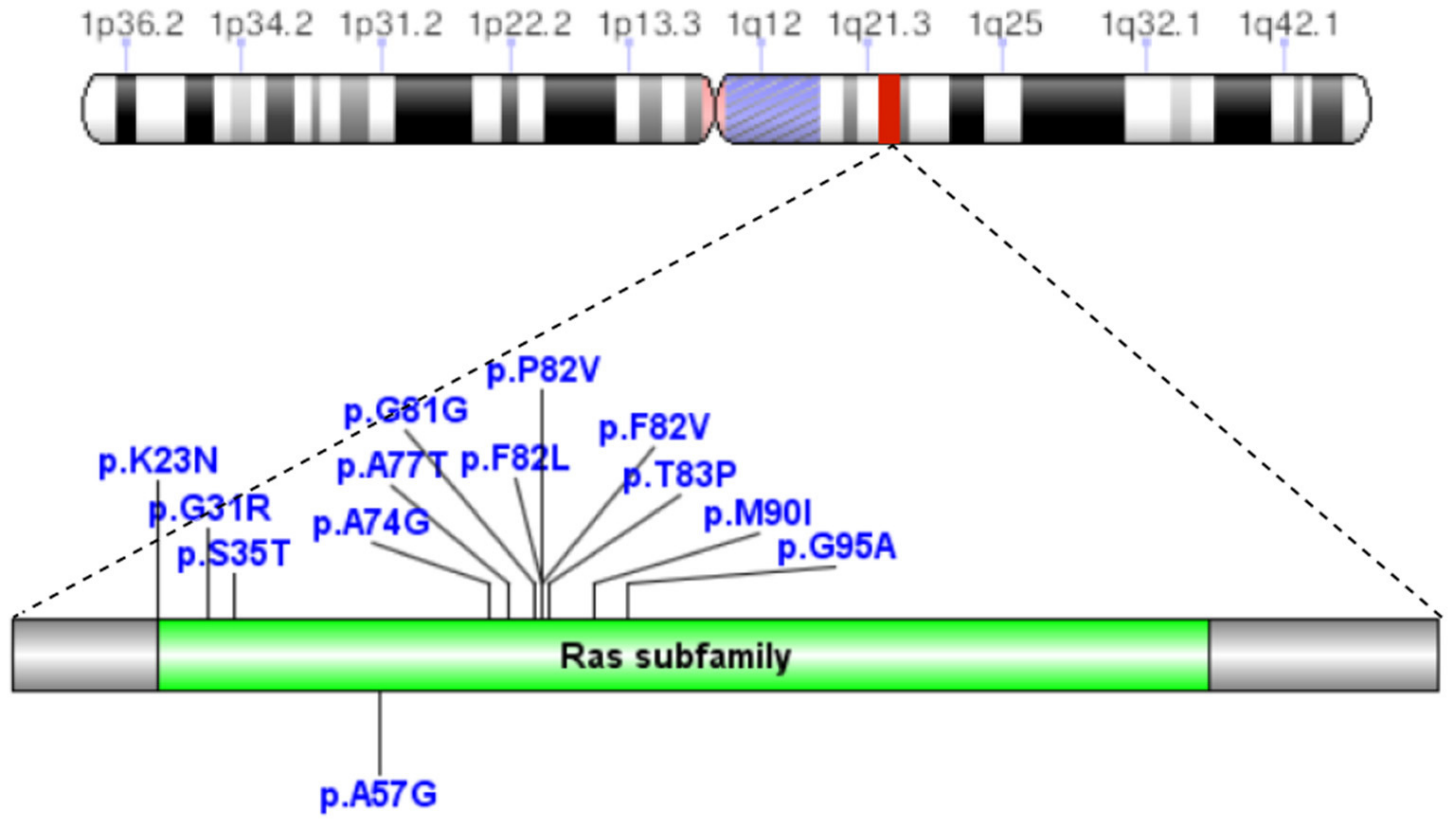
In 41 cases of NS caused by *RIT1* gene mutations, the mutation loci were p.A57G, p.G95A, p.F82L, p.P82V, p.F82V, p.M90I, p.S35T, p.G31R, p.K23N, p.T83P, p.A77T, p.A74G, and p.Glu81Gly (p.G81G).

**Figure 3:**
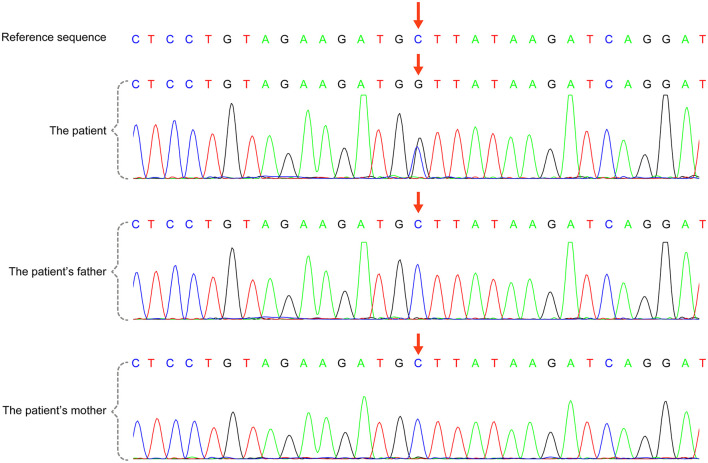
*RIT1* gene mutation in the case reported herein, validated by Sanger sequencing. There was a heterozygous variant in the *RIT1* gene in the child [chr1:c.170(exon4)C >G,P.A57G (NM-006912)], and the variant was not detected in his parents.

A total of 41 cases in 13 boys and 28 girls were retrieved. Fourteen cases were premature births. The age at diagnosis was 4 days to 18 years of age, respectively, and 10 cases were diagnosed at 0–1 years of age. Five cases had a familial genetic background, 18 cases had *de novo* mutation, and 18 cases had an unclear genetic background. The mutation sites included p.A57G (*n* = 13), p.G95A (*n* = 7), p.F82L (*n* = 5), p.P82V (*n* = 1), p.F82V (*n* = 2), p.M90I (*n* = 4), p.S35T (*n* = 3), and each one case of p.G31R, p.K23N, p.T83P, p.A77T, p.A74G, and p.Glu81Gly (p.G81G). There were two cases of death: one with p.A74G and the other with p.G81G (Table 1). Figure 1 shows a photograph of the child reported herein at 1.5 years of age. The distribution of the mutation loci of the *RIT1* gene in the 41 cases is shown in Figure 2. Validation of the *RIT1* mutation in the pedigree of our patient, determined by Sanger sequencing, is shown in Figure 3.

**Table 1:** Related literature and general clinical data of Noonan syndrome cases associated with *RIT1* gene mutation.

**Literature**	**Clinical Data**				
	**Sex (M/F)**	**Age at diagnosis(y)**	***RIT1* gene mutation locus**	**Genetic background**	**Outcome**
Chen et al. (2) (*n* = 3)	1/2	2–18	p.F82V, p.A57G, p.G95A	*De novo*	Alive
Gos et al. (25) (*n* = 4)	0/4	5.5–17.5	p.P82V, p.M90I, p.G95A	Unknown	Alive
Kouz et al. (5) (*n* = 25)	7/18	2 m to 16 y	p.A57G, p.G95A, p.F82L, p.F82V, p.M90I, p.S35T, p.G31R, p.K23N, p.T83P, p.A77T.	5, Inherited; 7, De novo; 13, unkown	Alive
Koenighofer et al. (9) (*n* = 2)	1/1	2–15	p.A57G, p.M90I	*De novo*	Alive
Ramond et al. (21) (*n* = 1)	1/0	3	p.G81G	*De novo*	Death
Chen et al. (26) (*n* = 4)	1/3	2.4 m to 1.5 Y	p.A57G, p.M90I	*De novo*	Alive
Safwat et al. (19) (*n* = 1)	1/0	4 d	p.A74G	Unknown	Death
The case we reported (*n* = 1)	1/0	1.5 m	p.A57G	*De novo*	Alive

### Clinical phenotypes

**Prenatal care:** Among the 33 cases with prenatal examination records, the overall rate of abnormalities was 63.63%, including 39.39% with cervical edema, 36.36% with polyhydramnios, 12.21% with fetal hydrops, 21.21% with suspected cardiac developmental defects and two cases of renal developmental malformation.

**Postnatal clinical features:** Clinical signs after birth included typical craniofacial deformities in 78.38% (29/37), manifested as ptosis (24/35); some patients had other signs such as low-set ears, a triangular face and a high palate arch. Abnormal skin and hair, including curly hair, hyperkeratosis, hemangioma and multiple moles, were observed in 15/34 patients. In addition, 19/35 patients had webbed necks, and 11/33 patients had thoracic dysplasia. One case had scoliosis, one case had a submandibular bone tumor, and one case had immature bone marrow cells. Lymphatic dysplasia was found in 54.29% (19/35) of patients, and lymphedema was found in 34.29% (12/35) of patients, including five patients with neonatal lymphedema, and 11/35 patients had chylothorax. In addition, urinary system abnormalities were found in 29.27% (12/41) of the patients. Among nine boys with clinical records, five had cryptorchidism (5/9). Eight cases had kidney abnormalities, including three cases of renal dysplasia (3/8), two cases of hydronephrosis (2/8), one case of renal tumor (1/8) and two cases of renal injury (2/8). Long-term follow up indicated that 13/32 patients had short stature, 14/28 patients had motor retardation, and 12/23 patients had learning disability.

**Cardiac developmental abnormalities:** A total of 87.80% of patients with NS had cardiovascular developmental or conduction abnormalities. Among them, 58.53% of patients had hypertrophic cardiomyopathy (HCM), 34.15% of patients had pulmonary artery or pulmonary valve stenosis (PVS), and 19.51% of patients had both HCM and PVS. Supraventricular tachycardia was found in 48.78% of patients, and one case had ventricular tachycardia. HCM with arrhythmia was present in 29.27% of the patients. A total of 46.34% of patients with *RIT1*-related NS had atrial septal defects (ASD), and some patients had ventricular septal defects (VSD), patent ductus arteriosus (PDA), and developmental malformations of the mitral valve, tricuspid valve and coronary artery. There were two deaths: one sudden death due to left main coronary artery atresia with myocardial ischemia, and one death due to moderate-severe PVS with ventricular tachycardia. The phenotypes and incidence rates of cardiovascular diseases associated with various mutation loci of the *RIT1* gene are shown in Table 2.

**Table 2:** Phenotypes and incidence of cardiovascular diseases associated with mutations of the *RIT1* gene at various positions.

**Diseases**	**Position** ** Abnormal rate (*n* = 41%)**	**57** ** (*n* = 13%)**	**95** ** (*n*= 7%)**	**82** ** (*n* = 8%)**	**90** ** (*n* = 4%)**	**Others** ** (*n* = 9%)**
HCM	58.53	84.62	0	75.00	75.00	44.44
PVS	34.15	38.46	42.86	25.00	75.00	11.11
HCM+PVS	19.51	30.77	0	12.50	75.00	0
PVST	48.78	46.15	42.86	62.50	50.00	66.67
HCM+PVST	29.27	38.46	0	50.00	25.00	22.22
ASD	46.34	53.85	42.86	62.50	50.00	22.22
VSD	21.95	41.67	16.67	12.50	25.00	11.11
PDA, valve stenosis	24.39	23.08	0	50.00	50.00	11.11

## Discussion

NS is a group of clinical syndromes caused by mutations in the genes encoding the RAS/mitogen-activated protein kinase (RAS/MAPK) signaling pathway. The three most commonly mutated genes are *PTPN11* (MIM 176876), *SOS1* (MIM 182530), and *RAF1* (MIM 164760), and the NS incidence attributed to their mutations is approximately 50, 20, and 5%, respectively. *De novo RIT1* mutation can also cause NS, with similar frequencies of RAF1 mutation accounting for 5% of cases (4). The *RIT1* gene encodes a small GTPase of the RAS subfamily and shares more than 50% sequence identity with the RAS protein. The RIT1 gene is located on the long arm of chromosome 1 and consists of six exons, mainly G1, G2, G3, G4, and G5. Mutations in the RIT1 gene can induce excessive activation of ELK1, a transcription factor regulating the expression of genes involved in cell growth and proliferation, apoptosis, tissue remodeling and angiogenesis.

Similarly to NS due to other genetic backgrounds, NS caused by *RIT1* mutations shows abnormal prenatal examination findings, mainly involving the cardiovascular and lymphatic system. Our literature review indicated that prenatal abnormalities were frequent: 63.63% (21/33) of patients had fetal neck edema, which was the most common abnormality, followed by polyhydramnios, cardiac developmental defects, pleural effusion and fetal edema. Previous studies (5) have reported an incidence of fetal neck edema of 32% in NS caused by mutations of the *PTPN11* gene, the most commonly mutated gene in NS. The incidence of fetal neck edema was 39.39% in *RIT1* mutation associated NS, a value slightly higher than that for *PTPN11*.

Our study suggested the typical facial features in patients with NS, such as a triangular face, antimongoloid slant of the palpebral fissure, ptosis, broad nasal bridge, low-set ears and webbed neck, are also common in NS caused by *RIT1* mutations, particularly ptosis, webbed neck and thoracic deformities. Previous studies (6, 7) have shown that with increasing age, facial deformities in some patients tend to improve and become difficult to detect. Our case showed unilateral ptosis in the neonatal period, but the degree of ptosis gradually decreased with age, thus suggesting that some facial deformities may have heterogeneous phenotypes with age.

More than 80% of patients with NS have abnormal cardiovascular development, including HCM, PVS, ASD and VSD, and some have complications of multiple malformations. PVS accounts for 60–70% of cardiovascular disease in NS, followed by HCM (20–30%). ASD (10–30%), atrioventricular canal defects (5–15%) and VSD (5–10%) are less common (8). We found that 87.80% of patients with *RIT1*-related NS had cardiac structural abnormalities, a value higher than the 79% in patients with *PTPN11*-related NS (9).

NS is associated with high genetic heterogeneity, and various causative genes are associated with different heart diseases and incidence rates. PVS is a common cardiovascular disease in NS; 60% of cases are mild, approximately 10% are moderate, and approximately 30% are severe. Mild PVS cases generally do not require intervention, whereas 50 and 100% of moderate and severe PVS, respectively, receive intervention (10). The incidence of PVS caused by different mutant genes also varies: that for *PTPN11* is 65%, that for *SOS1* is 70%, and that for *RAF1* is 21% (11). Our study showed that the incidence of PVS in *RIT1* related NS is 34.15%, thus suggesting that *RIT1* is also a major mutant gene in NS with PVS.

The RAS/MAPK signaling pathway plays roles in the regulation of myocardial cell division, growth, differentiation, aging and other signal transduction processes. NS is a common cause of HCM in RASopathies, and the incidence of NS-HCM is approximately 20–23% (12). However, the incidence of NS-HCM varies with mutated genes, and is approximately 20–23% for *PTPN11*, 18% for *SOS1*, 65% for *RAF1* and 36% for *RIT1* (11). In previous reports, *RIT1* has been found to be responsible for up to 70% of HCM cases (13). In our study, 58.53% of the patients with *RIT1* mutation-related NS had HCM, a value higher than the incidence of HCM caused by *PTPN1* and *SOS1* mutations.NS-HCM has an early age of onset, with an average age of diagnosis of 6 months (14), and has a mortality rate as high as 22% before 1 year of age (15). However, compared with sarcomere hypertrophic cardiomyopathy, NS-HCM has an overall low childhood mortality, delayed myocardial hypertrophy and low risk of malignant arrhythmias (16). Our literature review revealed seven cases diagnosed with hypertrophic cardiomyopathy before the age of 7 months (Table 3), all with complications of PVS. In the child reported herein, ventricular septal hypertrophy was present at the age of 1.5 months, but signs of hypertrophy disappeared at the age of 7 months, on the basis of echocardiography, thus suggesting a dynamic process of NS-HCM. Therefore, for the heart health management of patients with NS, some researchers recommend annual echocardiography before 3 years of age, and re-evaluation at 5 and 10 years of age (8).

**Table 3:** Echocardiographic images and data recordings from NS patients.

**9 days old**	**55 days old**	**7 months old**
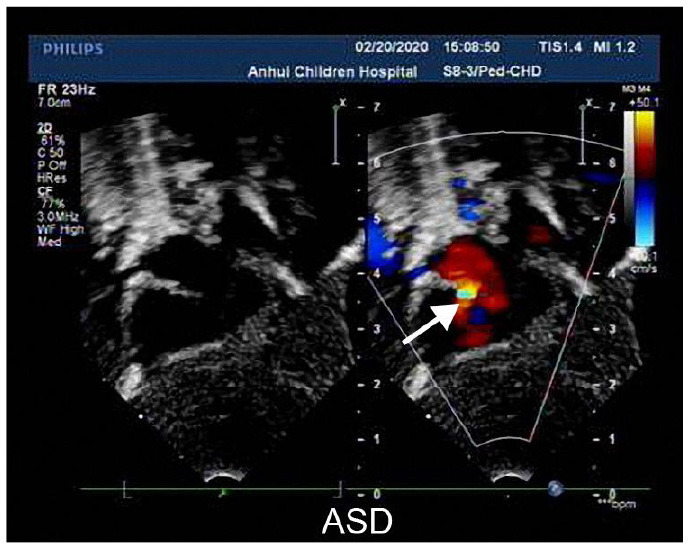	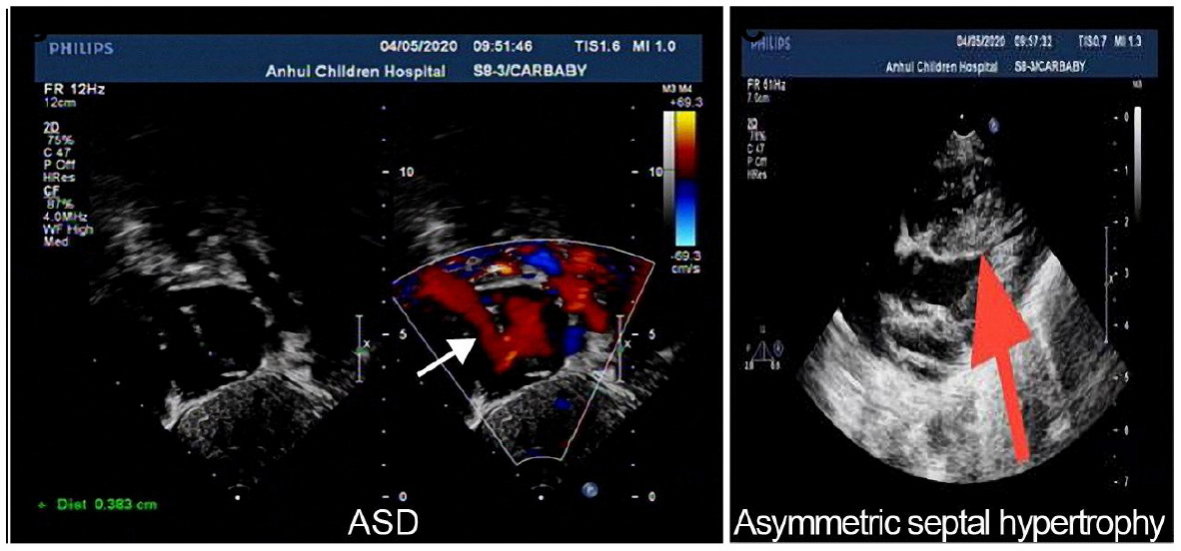	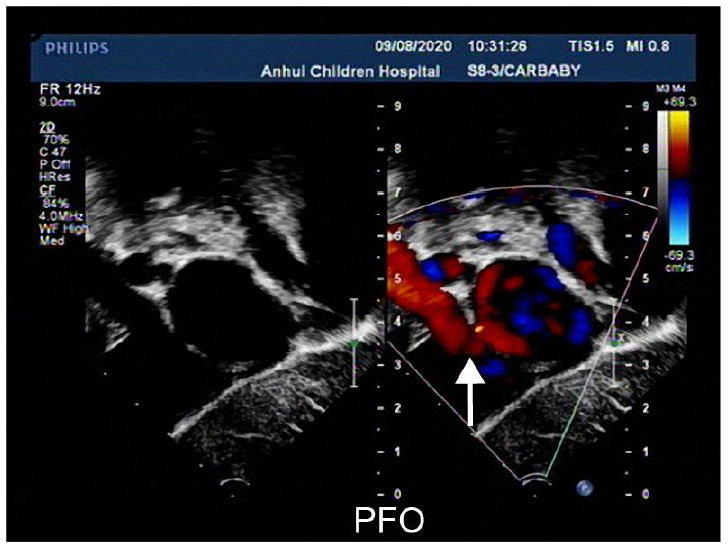
M-mode echocardiography: LVDD 1.8, LVDS 1.1 EF74%, FS40%, Two-dimensional echocardiography: ASD(secundum type,0.36cm), PDA is small, two dimensional echocardiography is not clear. Doppler echocardiography: ASD L → R(+), PDA L → R a small amount of shunt, MR(-), TR(+)mild regurgitation, PI(+)mild regurgitation, AI(-)	M-mode echocardiography: LVDD 1.7, LVDS 0.9, EF81%, FS47%; ASD(secundum type, 0.38cm), the ventricular septum of the heart was thickened, and the thicker middle part was 1.5cm,PDA is small, two dimensional Doppler echocardiography: LVOT2.7m/s, MPA2.1m/s, LPA2.6m/s PDA L → R a small amount of shunt,ASD L → R, MR(-),TR(+)mild regurgitation, PI(+)mild regurgitation,AI(-)	M-mode echocardiography: LVDD 2.2, LVDS 1.2 EF78%,FS44%, PFO Doppler echocardiography: AAO 1.0m/s,MPA3.0m/s PFO L → R(+), MR(-), TR(+)mild regurgitation, PI(+)mild regurgitation AI(-)

Mutations in different loci of the same gene result in different cardiac pathological changes. In 41 patients with NS associated with *RIT1* gene mutations, the common amino acid substitution positions included 57 (13/41), 82 (8/41), 95 (7/41), and 90 (4/41), accounting for 78.05% of all mutations. A total of 13 cases had replacement of alanine by glycine at the 57th amino acid (p.A57G), and were associated with the highest likelihood of developing HCM and a relatively low risk of PVS. In contrast, the incidence of heart disease, particularly HCM, was lower in the seven patients with p.G95A mutation (replacement of alanine by glycine at the 95th amino acid). Four patients with p.M90I mutation (replacement of methionine by leucine at the 90th amino acid) had HCM with PVS. Some studies (17) have indicated that p.A57G was located in the switch I region, whereas p.F82L, p.M90I, and p.G95A were located in the switch II region. *RIT1* mutant protein can regulate actin formation by enhancing complex formation among RIT1, RAC1/CDC42, and PAK1 (17). In animal experiments, Shingo et al. have found that Rit1 A57G/+ mice show cardiac hypertrophy, cell proliferation, and more pronounced fibrosis, but do not show cardiomyocyte hypertrophy and diminished cardiac function. In addition, Rit1 A57G/+ mice are highly susceptible to sympathetic stimulation and consequently substantial cardiac fibrosis (18). In the present study, we retrieved three cases with translocation of T35 in the *RIT1* gene. T35 in RAS protein directly binds magnesium ions and contacts catalytic water molecules. When the threonine residue is mutated, its catalytic effect is influenced, thus decreasing GTPase activity. No relevant clinical reports have been published to date, and the mechanism remains to be further studied.

Among RAS/MAPK pathway mutation diseases, Costello syndrome has a clear correlation with multifocal atrial tachycardia, but reports on supraventricular tachycardia in NS are limited. Our study identified 22 cases of paroxysmal supraventricular tachycardia, 12 accompanied by HCM and ten not accompanied by HCM, thereby suggesting no clear relationship between supraventricular arrhythmia and HCM. This finding is consistent with those from a study by Levin et al. (19). Our study also found one patient (20) with moderate to severe PVS who died of ventricular tachycardia, which was considered to be associated with genetic heterogeneity, after balloon dilation.

The incidence of ASD, another cardiac malformation, was higher in patients with *RIT1* mutation (46.34%) than *PTPN11* (10%), *SOS1* (14.5%), and *RAF1* (19.6%) mutations (11). The incidence of VSD in patients with *RIT1* mutation (21.95%) was similar to that associated with other gene mutations. According to one study (21), approximately 30% of patients with RASopathies have complications of coronary artery dysplasia. In our study, one patient (22) had left main coronary artery atresia which led to sudden myocardial ischemia death.

In addition to facial abnormalities, curly hair, dysplasia of the sternum, spine and kidney, and growth retardation are also common in patients with *RIT1*-mutated NS. An animal study has introduced *RIT1* mutations (c.236A>T [p.Gln79Leu], c.242A>G [p.Glu81Gly], and c.284G>C [p.Gly95Ala]) into zebrafish zygote embryo cells and observed craniofacial abnormalities, pericardial edema, yolk sac elongation, skeletal and spinal abnormalities in embryos and severe developmental delay in approximately 7% of RIT1-mutated embryos (9). Animal experiments have shown that *RIT1* gene mutations could cause NS-related phenotypes. One case of NS has been reported to have a complication of submandibular tumor (5), and one case has been reported to have a complication of bone marrow immature mononucleosis (20). Current studies (21, 23, 24) have indicated that somatic mutation of *RIT1* was associated with myeloid malignancies and lung adenocarcinomas. Mutation sites are concentrated around the 79th glutamine in the switch II region (p.A77P, p.G81G, p.P82L/V, and p.M90I), and mutant proteins can induce cell transformation.

In summary, *RIT1* gene mutation-related NS had a high rate of abnormalities found in prenatal examinations. Most cases were characterized by typical facial features after birth, and were often associated with cardiac developmental malformations and conduction disorders. Cardiovascular system abnormalities caused by mutations at various sites showed high genetic heterogeneity. Some patients had a risk of long-term growth retardation and cancer, and required long-term follow-up.

## Data availability statement

The original contributions presented in the study are included in the article/supplementary material, further inquiries can be directed to the corresponding author.

## Ethics statement

Written informed consent was obtained from the minor(s)' legal guardian/next of kin for the publication of any potentially identifiable images or data included in this article.

## Author contributions

LD and PZ designed the study and wrote the manuscript. LD, PZ, YK, LW, YW, and QQ investigated and provided the clinical data. All authors contributed to the article and approved the submitted version.

## Conflict of interest

The authors declare that the research was conducted in the absence of any commercial or financial relationships that could be construed as a potential conflict of interest.

## Publisher's note

All claims expressed in this article are solely those of the authors and do not necessarily represent those of their affiliated organizations, or those of the publisher, the editors and the reviewers. Any product that may be evaluated in this article, or claim that may be made by its manufacturer, is not guaranteed or endorsed by the publisher.
